# Translation and validation of the 5-Item Dry Eye Questionnaire into Portuguese

**DOI:** 10.5935/0004-2749.2022-0197

**Published:** 2022-10-19

**Authors:** Luísa Grave Gross, Maria Eugênia Pozzebon, Arthur Pinheiro Favarato, Gabriel Ayub, Mônica Alves

**Affiliations:** 1 Department of Ophthalmology, Faculdade de Ciências Médicas, Universidade Estadual de Campinas, Campinas, SP, Brazil

**Keywords:** Dry eye syndromes, Diagnostic techniques, ophthal-mological, Surveys and questionnaires, Translations, Síndromes do olho seco, Técnicas de diagnóstico oftalmológico, Inquéritos e questionários, Traduções

## Abstract

**Purpose:**

Dry eye disease is a multifactorial disease that is very common in clinical ophthalmic practice. The use of validated dry eye questionnaires makes it possible to screen this disease in the general population and assess the prevalence of symptoms and frequency of diagnosis, allowing early and appropriate treatment for this condition. The 5-Item Dry Eye Questionnaire (DEQ-5) is a tool that is used to assess the frequency and intensity of ocular discomfort and dryness and the frequency of tearing, which has already been validated in English and Spanish, but not in Portuguese. The aim of this study is to translate and validate the DEQ-5 to Portuguese.

**Methods:**

The DEQ-5 was used, consisting of five simple and direct questions: two questions for ocular discomfort, two for ocular dryness, and one for tearing. The initial translation of the English version of the questionnaire into Portuguese was conducted by two Portuguese native-speaking translators, followed by an evaluation and compilation of a single version by an interdisciplinary committee of the translated versions. Furthermore, this version was translated back into English by two individuals whose first language was English, followed by the evaluation and comparison with the original version in English by the same interdisciplinary committee. Afterwards, the final version of the questionnaire was administered to 31 volunteers at two different times.

**Results:**

The interobserver reliability of the five questions ranged from 0.584-0.813, and the Pearson correlation from 0.755-0.935, with a p-value of <0.0001. Internal consistency was α=0.887. All questions had moderate to high agreement.

**Conclusions:**

The statistical analysis of the collected data found excellent concordance rates (moderate to high for all analyzed questions), allowing the use of the Portuguese version of DEQ-5 in research as a screening test for dry eye disease and tool used to monitor the symptoms.

## Abstract (pt)

**Ojetivos:**

O olho seco é uma doença multifatorial, muito comum na prática clínica oftalmológica. A utilização de questionários validados de sintomas de olho seco permite rastrear de maneira mais objetiva essa doença na população geral, avaliar a prevalência dos sintomas e frequência de diagnóstico, permitindo o tratamento precoce e adequado dessa condição. O 5-Item Dry Eye Questionnaire (DEQ-5) é um questionário em inglês que avalia a frequência e a intensidade do desconforto e ressecamento ocular e a frequência do lacrimejamento, já validado no inglês e espanhol, porém não no português até o presente momento. O objetivo deste estudo foi traduzir e validar o questionário DEQ-5 para a língua portuguesa.

**Métodos:**

Foi utilizado o questionário DEQ-5, composto por 5 perguntas simples e diretas - 2 relacionadas ao desconforto ocular, 2 ao ressecamento ocular e 1 ao lacrimejamento. Foi realizada a tradução inicial da versão em inglês para o português por dois tradutores de língua materna portuguesa, seguido de uma avaliação e compilação de uma versão única por um comitê interdisciplinar das versões traduzidas. Em seguida, tal versão foi traduzida de volta para o inglês por 2 indivíduos que possuíam o inglês como primeira língua, seguido de avaliação e comparação com a versão original em língua inglesa pelo mesmo comitê interdisciplinar. Após, a versão final do questionário foi aplicada em 31 voluntários em dois momentos differentes.

**Resultados:**

A confiabilidade interobservador das 5 perguntas variou de 0,584-0,813 e a correlação de Pearson de 0.755-0.935, sendo o p-valor <0,0001 em todas elas. A consistência interna foi α=0,887. Todas as perguntas apresentaram concordância de moderada a alta.

**Conclusões:**

A análise estatística dos dados coletados encontrou ótimos índices de concordância - moderada a alta para todas as questões analisadas -, permitindo o uso do DEQ-5 na língua portuguesa em pesquisas e também como forma de triagem para doença de olho seco e acompanhamento dos sintomas.

## INTRODUCTION

Studies in the medical field have used questionnaires to quantify and qualify symptoms in order to estimate the prevalence of the disease and correlate its impact on the quality of life of patients^(1, 2^, ^3)^. For this purpose, the questionnaires can have two different origins: the first is the creation of a questionnaire in the desired language for the study population, and the second is the translation and validation of an existing questionnaire into another language for another population group but which can be widely applied. The second option has more advantages, as it saves time and resources and because it offers the possibility of comparing the results obtained in different populations^(4)^.

Questionnaires are important to measure the patients’ symptoms more objectively, as many concepts used in the medical field are individual and subjective. Thus, the data obtained can be compared between different researchers or even by the researcher at different stages of the disease of the same patient. The obtained results also allow more detailed studies on the effect of the treatments to be conducted, providing comparison analyses of the impact on the patient’s quality of life^(5)^.

Dry eye disease is a very common eye condition in clinical ophthalmic practice^(5,6)^. It is a multifactorial disease that is associated with several intrinsic risk factors, such as aging, menopause, and autoimmune diseases, as well as extrinsic factors, such as environmental exposure, medications of topical and systemic use and the use of contact lenses. The prevalence of dry eye disease in the general population has been reported to be higher in the elderly and women, but based on the large number of associated conditions, it can also be observed in young patients. According to the last consensus held on dry eye disease, the prevalence in different populations is still not completely known and presents itself in a very variable way, indicating the need for more population studies to better understand these numbers and associated risk factors^(6, 7^, ^8)^. Although this disease has no cure, early diagnosis allows treatment to be started, alleviating signs and symptoms and reducing complications. The impact of dry eye disease on the quality of life and vision of patients is closely related to its severity, etiology, and associated environmental factors.

While the old definition of dry eye disease emphasized signs of inflammation on the ocular surface and dysfunction of the lacrimal system, the definition proposed in 2007 by the Dry Eye Workshop (DEWS) placed the symptoms more prominently in the disease. Thus, the assessment of dry eye symptoms via questionnaires plays an important role in the diagnosis, treatment, and monitoring of the disease^(9)^ in addition to making it possible to compare the results of different studies^(5,10^, ^11, 12)^.

According to DEWS 2007, a total of 14 questionnaires are validated in English for the assessment of dry eye disease ^(5)^. Recently, DEWS II 2017 recognized that the DEQ-5 is an adequate tool as it is concise and allows the distinction of patients with and without dry eye disease and, in the group of patients with dry eye disease, those with and without Sjögren’s syndrome (SS), a disease that is characterized by severe dry eyes (score of >6 is suspected of dry eye and score of >12 is suspected of SS)^(8,9,13)^. This questionnaire consists of five simple and direct questions from the Dry Eye Questionnaire, in which two questions measure ocular discomfort, two for ocular dryness, and one for tearing, and can be used as a form of screening to help the treating physician determine which patients should undergo a detailed investigation for dry eye.

The aim of this study is to translate and validate the DEQ-5 into Portuguese, which assesses the frequency and intensity of ocular discomfort and dryness and the frequency of tearing. This questionnaire was validated in the English language and translated and validated in Spanish^(9,12)^, but not in Portuguese.

## METHODS

This is an observational, cross-sectional study, which was conducted at the Department of Ophthalmology, *Hospital de Clínicas, Universidade Estadual de Campinas* (HC-UNICAMP), Brazil, after the approval by the Research Ethics Committee (CAAE 38021820.3.0000.5404).

The DEQ-5 was used, consisting of five simple and direct questions in which two questions measure ocular discomfort, two for ocular dryness, and one for tearing.

With regard to Questions 1a, 2a, and 3, the answer options are five: never, rarely, sometimes, frequently, and constantly. The answer options for Questions 1b and 2b are six; the intensity of the symptoms are measured on a scale from 0 to 5, with “never having presented the symptoms” being equivalent to 0 and “very intense” to 5.

In order to obtain the translation and cross-cultural validation of the original English version of the DEQ-5 into Portuguese, a process proposed by Beaton et al. was applied^(4,14)^. First, the initial translation and cross-cultural adaptation of the English version into Portuguese was conducted by two independent translators whose native language is Portuguese, followed by an evaluation of the translated version by an interdisciplinary committee composed of two ophthalmologists and two ophthalmology residents who compiled a single version of the questionnaire in Portuguese. Moreover, the Portuguese version of the questionnaire was translated back into English by two independent individuals whose native language is English, followed by the evaluation and comparison of the original English version by the same interdisciplinary committee. Afterwards, the final Portuguese version of the questionnaire was applied by two independent observers in a sample of 31 people at two different times, separated by an interval of 2 to 5 days. The administered questionnaires were completed by the volunteers alone without the assistance of the observers. This group consisted of residents, fellows, assistant physicians, and nursing staff from the Department of Ophthalmology at UNICAMP who signed the informed consent form after being briefed about the study. Finally, the statistical analysis of the responses was conducted to determine the correlation values and kappa agreement.

The internal consistency (Cronbach’s alpha) was calculated to assess the intercorrelation of the questionnaire items, ranging from 0 to 1, with a value of >0.7 being considered adequate^(15)^. Pearson’s correlation (R) and the interobserver reliability (Cohen’s Kappa (κ)) were calculated (ranging from 0 to 1) whose values were interpreted as very low agreement (<0.2); low agreement (0.2-0.4); moderate agreement (0.4-0.6); good agreement (0.6-0.8); and excellent agreement (>0.8)^(4,15)^. A p-value of less than 0.05 was considered statistically significant. The analysis was performed with the Statistical Package for Social Sciences (SPSS) (IBM Corporation, Armon NY, USA, version 22.0).

**Figure 1:**
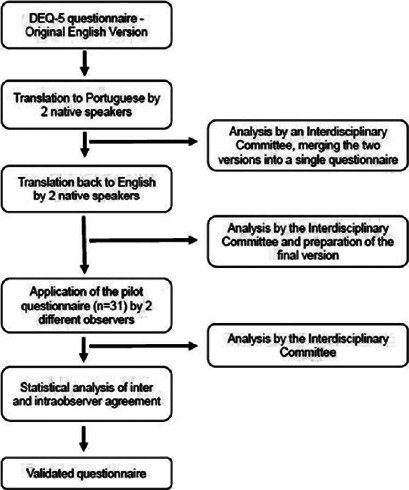
Study design.

Figure 1 shows the flowchart of the proposed study design.

## RESULTS

**Figure 2:**
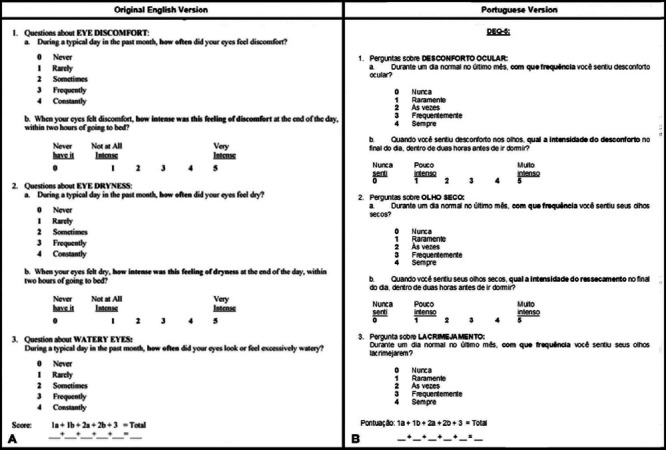
A) Original DEQ-5 version in English^(9)^. B) Translated and validated version in Portuguese.

Thirty-one resident physicians, fellows, assistant physicians, and nursing staff from the Department of Ophthalmology at UNICAMP completed the test-retest process of the translated questionnaire. No relevant difficulties were encountered in the process of translation and the application of the questionnaires. Figure 2 shows the Portuguese and original English versions of the DEQ-5.

Table 1 shows the interobserver reliability (κ) and the Pearson correlation (R) with their respective p-values. The interobserver reliability of the five questions ranged from 0.584-0.813 and the Pearson correlation from 0.7550.935, with a p-value of <0.0001. Internal consistency was α=0.887. All questions had moderate to high agreement.

**Table 1:** Interobserver reliability, Pearson correlation, and p-value of each DEQ-5 item

Question	Interobserver reliability (κ)	Pearson correlation (R)	p-value
1A	0.813	0.935	<0.0001
1B	0.667	0.848	<0.0001
2A	0.691	0.902	<0.0001
2B	0.584	0.889	<0.0001
3	0.688	0.755	<0.0001

## DISCUSSION

Dry eye disease is a multifactorial disease that impacts the quality of life and vision of carrier patients, as tear film instability and corneal irregularities resulting from keratitis lead to changes in the optical quality of the corneal-tear film interface^(5)^. It is an extremely common condition that is associated with different risk and causal factors as well as variable intensities of ocular surface homeostasis involvement and symptoms reported by the patients. It is considered as a symptomatic disease and the quantification of the associated symptoms is important in the diagnostic investigation and the patients’ follow-up. The main consensus in the area suggests that screening for symptoms and the assessment of risk factors should be performed as the first step in the disease investigation.

Thus, the assessment of dry eye symptoms using a questionnaire plays an important role in the diagnosis, treatment, and monitoring of this disease. The DEQ-5 is a short, simple, and self-administered questionnaire that distinguishes patients with and without dry eye disease and, in the group of patients with dry eye disease, those with and without Sjögren’s Syndrome-an autoimmune disease that constitutes the main cause of dry eye due to water deficiency^(9)^.

The use of the translated questionnaires, especially in the context of research, should be conducted after the validation of such translation and not simply translate and use a certain tool in another linguistic context, as it would lead to inaccurate results, as simple as the questions may seem. Thus, the translated tools validated in a certain language and sociocultural and temporal context allow the results to be compared with those obtained in the questionnaires in other languages and in the original language of development of the questionnaire^(4)^.

The DEQ-5 was validated in its original English version by Chalmers et al. in 2010 and validated in Spanish by Martinez et al. in 2019^(9,12)^. The latter group evaluated the accuracy of the DEQ-5, indicating a sensitivity of 76% and a specificity of 31% in the score above or equal to 6 points^(12)^. Recently, the DEQ-5 was compared with the OSDI questionnaire by Akowuah et al. in 2021 who concluded that the DEQ-5 is a valid tool for both the assessment of symptoms of dry eye and its use in clinical and epidemiological studies. The same group found maximum values of sensitivity (71.2%) and specificity (82.7%) in the score of 5.5 points on the DEQ-5. This correlates with the diagnosis of suspected dry eye in the score above or equal to 6 points in this questionnaire. They also emphasized that the OSDI is the most used questionnaire for assessing dry eye symptoms. However, it only assesses the frequency of such symptoms and their effect on daily activities, while the DEQ-5 assesses not only the frequency but also the intensity of such symptoms^(16)^. However, to date, this questionnaire had not been validated in the Portuguese language, making it hard for it to be considered an accurate tool in the clinical and scientific perspective.

In this study, we performed the translation and cross-cultural validation of the original English version of the DEQ-5 into Portuguese, following the three-phase process proposed by Beaton et al.^(4,14)^. Our study was structured in recognized translation and validation protocols of the diagnostic tools for quantifying symptoms. The translation process involved participants native in the two languages and was conducted without relevant difficulties as it was a direct and concise text. The validation process included the participation of volunteers evaluated at different times according to protocol. The statistical analysis of the collected data found excellent agreement rates from moderate to high for all analyzed questions.

The translation and validation of the DEQ-5 into Portuguese will allow its use as a means of screening for dry eye disease and the monitoring of symptoms. Thus, this tool can be effectively used in studies, in addition to helping the assistant physician in determining which patients should undergo a detailed investigation of dry eye disease in the specialized clinical assessment routine, and as a tool to monitor the evolution of symptoms of patients undergoing treatment.
